# Functional consequences of the first reported mutations of the proto-oncogene PTTG1IP/PBF

**DOI:** 10.1530/ERC-16-0340

**Published:** 2017-07-04

**Authors:** W Imruetaicharoenchoke, A Fletcher, W Lu, R J Watkins, B Modasia, V L Poole, H R Nieto, R J Thompson, K Boelaert, M L Read, V E Smith, C J McCabe

**Affiliations:** 1 Institute of Metabolism and Systems ResearchUniversity of Birmingham, Birmingham, UK; 2 Centre for EndocrinologyDiabetes and Metabolism, Birmingham Health Partners, Birmingham, UK; 3 Department of SurgeryFaculty of Medicine Siriraj Hospital, Mahidol University, Bangkok, Thailand; 4 Institute of Cancer and Genomic SciencesUniversity of Birmingham, Birmingham, UK

**Keywords:** COSMIC, TCGA, PTTG1IP, mutation, proliferation

## Abstract

Pituitary tumor-transforming gene 1-binding factor (PTTG1IP; PBF) is a multifunctional glycoprotein, which is overexpressed in a wide range of tumours, and significantly associated with poorer oncological outcomes, such as early tumour recurrence, distant metastasis, extramural vascular invasion and decreased disease-specific survival. PBF transforms NIH 3T3 fibroblasts and induces tumours in nude mice, while mice harbouring transgenic thyroidal PBF expression show hyperplasia and macrofollicular lesions. Our assumption that PBF becomes an oncogene purely through increased expression has been challenged by the recent report of mutations in PBF within the Catalogue of Somatic Mutations in Cancer (COSMIC) database. We therefore sought to determine whether the first 10 PBF missense substitutions in human cancer might be oncogenic. Anisomycin half-life studies revealed that most mutations were associated with reduced protein stability compared to wild-type (WT) PBF. Proliferation assays narrowed our interest to two mutational events which significantly altered cell turnover: C51R and R140W. C51R was mainly confined to the endoplasmic reticulum while R140W was apparent in the Golgi apparatus. Both C51R and R140W lost the capacity to induce cellular migration and significantly reduced cell invasion. Colony formation and soft agar assays demonstrated that, in contrast to WT PBF, both mutants were unable to elicit significant colony formation or anchorage-independent growth. However, C51R and R140W retained the ability to repress radioiodide uptake, a functional hallmark of PBF. Our data reveal new insight into PBF function and confirm that, rather than being oncogenic, mutations in PBF are likely to be passenger effects, with overexpression of PBF the more important aetiological event in human cancer.

## Introduction

Only a minority of mutations are likely to confer a specific growth advantage *in vivo*, resulting in clonal expansion and tumour development (driver mutations), whilst the majority of substitutions reported in sequencing databases such as The Cancer Genome Atlas (TCGA) and the Catalogue of Somatic Mutations in Cancer (COSMIC) are likely to be passenger mutations (Greenman *et al*. 2007). Functional clarification is now urgently needed to discern the driver mutations which may represent new or refined diagnostic, prognostic or therapeutic markers for particular types of cancer, and to more fully understand the mechanisms responsible for tumour formation and progression.

We previously characterised *PBF* (pituitary tumor-transforming gene 1-binding factor; also known as *PTTG1IP* or *c21orf3*) as a proto-oncogene based on the transformation potential of PBF *in vitro* and its ability to induce tumours in xenograft models (Stratford *et al*. 2005). We and others have demonstrated that *PBF* is overexpressed in pituitary, thyroid, breast and colorectal tumours, and that upregulation of the gene is significantly associated with poorer oncological outcome in a range of tumours (McCabe *et al*. 2003, Stratford *et al*. 2005, Watkins *et al*. 2010, Hsueh *et al*. 2013, Read *et al*. 2016). For instance, papillary thyroid cancer patients with high PBF expression at the time of diagnosis show decreased disease-specific survival compared to those who have low PBF expression (Hsueh *et al*. 2013). Colorectal tumours which show extramural vascular invasion have significantly higher PBF expression (Read *et al*. 2016), and high PBF promoter activity is associated with poorer clinical outcome and increased metastatic risk in breast cancer (Xiang *et al*. 2012). However, whilst transgenic PBF mice demonstrated increased AKT phosphorylation, thyroid gland enlargement, hyperplasia and macrofollicular lesions, they did not routinely develop tumours (Read *et al*. 2011). The role of PBF in tumourigenesis is therefore unclear.

PBF is ubiquitously expressed and highly conserved (Smith *et al*. 2011). It interacts with a range of proteins impacting on various cellular functions. The interaction with the human securin, PTTG, from which it gained its name, mediates the translocation of PTTG into the nucleus where it influences a number of cellular processes such as cell cycle regulation, genetic instability and gene transactivation (Vlotides *et al*. 2007). PBF also binds to the sodium iodide symporter (NIS) and monocarboxylate transporter 8 (MCT8), modulating their subcellular localisation, and regulating radioiodide uptake and thyroid hormone efflux, respectively (Smith *et al*. 2009, 2012, 2013, Read *et al*. 2011). Furthermore, overexpression of PBF increases p53 ubiquitination, resulting in dysregulated p53 cellular function (Read *et al*. 2014, 2016). Recent *in silico* efforts to identify driver genes amongst next generation sequencing data identified *PBF* as a putative oncogene (Melloni *et al*. 2014).

Proto-oncogenes generally become oncogenic either through mutation or overexpression leading to gain-of-function. The TCGA (Chin *et al*. 2011, Tomczak *et al*. 2015) and COSMIC (Forbes *et al*. 2008, 2011, 2015) databases are now revealing mutational changes in proto-oncogenes that are considered to be oncogenic through enhanced expression, opening the possibility that these genes may, in rare cases, also act as oncogenes through functional activation. We utilised PBF as a paradigm of this idea, assessing whether the first 10 reported mutations of PBF in COSMIC might have oncogenic gain-of-function. Given that PBF is implicated in the aetiology of multiple tumour types, we determined the influence of the first 10 reported mutations of PBF upon cellular proliferation, before progressing a subset of mutations to canonical assays of cellular invasion, migration and anchorage-independent growth. Our data shed new light on PBF function and suggest that PBF upregulation, rather than mutation, is more critical to human tumour development and progression.

## Materials and methods

### The Cancer Genome Atlas (TCGA)

The TCGA project (http://cancergenome.nih.gov/) comprises a genomic data analysis pipeline that has resulted in the mapping of genomic alterations in more than 11,000 human tumours across 33 types of cancer (Chin *et al*. 2011, Tomczak *et al*. 2015). Data were obtained using the cBioPortal for Cancer Genomics (http://www.cbioportal.org/) (Cerami *et al*. 2012, Gao *et al*. 2013).

### Catalogue of Somatic Mutations in Cancer (COSMIC) database

The COSMIC database (http://cancer.sanger.ac.uk/cosmic), started in 2004, describes somatic mutation information in human cancers (Forbes *et al*. 2008, 2011, 2015). Data are collated through manual curation of scientific literature describing mutations in known cancer genes selected from the Cancer Gene Census (http://cancer.sanger.ac.uk/cancergenome/projects/census/) and from large cancer genome datasets found in published literature and online data portals (Forbes *et al*. 2015). PBF mutation data were obtained from COSMIC in April 2013.

### Cell lines

NIH 3T3 murine fibroblast and MCF7 breast cells were obtained from the American Type Culture Collection (ATCC) and maintained in DMEM (Sigma) and RPMI 1640 medium (Thermo Fisher Scientific), respectively. TPC1 and SW1736 thyroid carcinoma cell lines were kindly supplied by Dr Rebecca Schweppe (University of Colorado) and maintained in RPMI 1640 medium (Thermo Fisher Scientific). COS-7 African green monkey kidney epithelial and HeLa human cervical carcinoma cancer cell lines were acquired from the European Collection of Authenticated Cell Cultures (ECACC, Porton Down, UK) and maintained in DMEM, high glucose (Sigma). All were supplemented with 10% foetal bovine serum (FBS; Thermo Fisher Scientific), penicillin (10^5^ U/L), and streptomycin (100 mg/L). As PBF is best characterised in thyroid and breast cancer, most of the experiments were performed using TPC1, SW1736 and MCF7 cells. Some experimental approaches required alternative or additional cell lines. For example, immunofluorescent determination of subcellular localisation was most effective in large, easily transfected cells such as HeLa cells and soft agar assays required stable, non-transformed NIH 3T3 cells.

### Plasmids and transfection

Plasmids containing full-length PBF cDNA with or without a C-terminal haemagglutinin (HA) tag have previously been described (Stratford *et al*. 2005). All 10 PBF mutations were recapitulated in both HA-tagged and untagged PBF using the QuikChange II XL Site-Directed Mutagenesis Kit (Stratagene). FLAG-tagged PBF was generated through the ligation of an oligo encoding the FLAG sequence (DYKDDDK) into the pcDNA3.1+_PBF plasmid. The FLAG tag lies within the protein after residue E34. Previous attempts to tag PBF at the N-terminal end were unsuccessful due to the presence of a cleavable signal peptide; to overcome this, the FLAG epitope lies downstream of the cleavage site. The C51R and R140W mutations were also introduced into FLAG-tagged PBF. The NIS cDNA was housed in the pcDNA3.1+ vector with a C-terminal MYC tag (Smith *et al*. 2009). For stable transfections, untagged wild-type (WT) and PBF mutant cDNAs were cloned into the pCI-neo vector (Promega). Transfections were performed with TransIT-LT1 reagent (Geneflow, Lichfield, UK) following the manufacturer’s protocol at a 3:1 reagent to DNA ratio and the experiments were performed after 24–48 h.

### Immunofluorescence staining

Immunofluorescence experiments were performed 48 h post-transfection in HeLa and MCF7 cells as described previously (Smith *et al*. 2009). Primary antibodies used included mouse monoclonal anti-HA.11 (1:200; BioLegend, San Diego, CA, USA), rabbit polyclonal anti-HA (Y-11) (Santa Cruz Biotechnology), mouse monoclonal anti-FLAG M2 (1:200; Sigma), rabbit polyclonal anti-PDI (1:200; Cell Signaling Technology), rabbit monoclonal anti-Golgin-97 (1:200; Cell Signaling Technology) and rabbit polyclonal anti-NIS ab104920 (1:500; Abcam). Cells were visualised using a Zeiss Axioplan fluorescent microscope (Zeiss) with a 100× objective.

### Western blotting

Cells were harvested in radioimmunoprecipitation assay (RIPA) (50 mM Tris–HCl, pH 7.4, 150 mM NaCl, 1% vol/vol IGEPAL CA-630, 6 mM sodium deoxycholate, 1 mM EDTA) with protease and phosphatase inhibitor cocktails (Sigma). Western blotting was performed as described previously (Smith *et al*. 2012). Proteins (30 µg) were separated by SDS-PAGE using a 15% acrylamide gel (12% for the detection of NIS). Primary antibodies used were mouse monoclonal anti-HA.11 (1:1000; BioLegend), mouse monoclonal anti-FLAG M2 (1:500; Sigma), anti-PBF antibody (made by Eurogentec (Seraing, Belgium) using the full-length PBF protein as an epitope), mouse monoclonal anti-MYC-Tag 9B11 (1:1000; Cell Signaling Technology) and mouse monoclonal β-actin AC-15 (1:10,000; Sigma).

### Half-life study

MCF7 cells were transfected in 6-well plates with vector only (VO), HA-tagged WT and mutant PBF, and FLAG-tagged WT and R140W PBF, and incubated for 48 h. The cells were treated with 5 µL anisomycin (Sigma) in 1 mL Opti-MEM serum-free media (Thermo Fisher Scientific) for 0, 12 and 24 h. Protein stability was determined through Western analysis.

### Sorting Intolerant From Tolerant (SIFT) assessment

SIFT (http://sift.jcvi.org/) is a software program that is used to predict whether an amino acid substitution impacts upon protein function. SIFT prediction is based on the degree of conservation of amino acid residues in closely related sequences collected through Position-Specific Iterative Basic Local Alignment Search Tool (PSI-BLAST) (Sim *et al*. 2012). An amino acid substitution that is predicted to have a phenotypic effect, with a SIFT score of less than 0.05, is classified as ‘damaging.’ A mutation that is unlikely to have a phenotypic effect (SIFT score >0.05) is considered ‘tolerated.’

### Glycosylation and dimerisation analysis

For deglycosylating PNGase F treatment, 15 µg protein lysate from COS-7 transfected with either PBF-HA or PBF-N45/54A-HA was denatured for 30 min at 37°C with 10× Glycoprotein Denaturing Buffer (NEB, Ipswich, MA, USA) followed by incubation with 1 µL PNGase F (NEB), 2 µL 10% NP40 and 2 µL 10× G7 buffer at 37°C overnight. Glycosylation status was determined through protein size discrimination by Western blotting. Cells were alternatively treated with the N-linked glycosylation inhibitor tunicamycin (1 µg/mL; Sigma) for 24 h prior to harvesting. The presence of a dimeric form of PBF ~50 kDa was determined through the omission of β-mercaptoethanol in the protein loading buffer. Dimerisation was also determined using proximity ligation assays (PLAs) which detect protein–protein interactions. HeLa cells were transfected with FLAG-PBF and PBF-HA, FLAG-PBF and VO, or PBF-HA and VO. After 48 h cells were fixed, permeabilised, blocked and incubated with primary antibody (mouse anti-FLAG and rabbit anti-HA) as performed in the immunofluorescent studies. The Duolink In Situ Kit (Olink, Uppsala, Sweden) was then used as per the manufacturer’s instructions. Images were obtained using the Zeiss confocal LSM 510 microscope ×40 objective.

### Cell proliferation assays

For the bromodeoxyuridine (BrdU) assay TPC1 and MCF7 cells were transfected with VO, untagged WT and mutant PBF. After 24 h, cells were reseeded into 96-well plates with 8 replicates per condition. The Cell Proliferation ELISA, BrdU (colorimetric) kit (Roche) was used a further 24 h later following the manufacturer’s protocol with a BrdU incubation time of 4 h.

For the 3-(4,5-dimethylthiazol-2-yl)-5-(3-carboxymethoxyphenyl)-2-(4-sulphophenyl)-2H-tetrazolium, inner salt (MTS) assay, 24 h post-transfection cells were reseeded into 96-well plates with 6 replicates per condition. The CellTiter 96 AQueous One Solution Cell Proliferation Assay (Promega) was used a further 24 h later following the manufacturer’s protocol with an MTS reagent incubation time of 4 h.

### 2D Boyden chamber invasion assays

Forty-eight hours post-transfection with VO, untagged WT PBF, C51R and R140W, TPC1 and MCF7 cells were switched to media containing 2% FBS and were incubated for 16 h. Cells were reseeded into Corning BioCoat Growth Factor Reduced Matrigel Invasion Chambers with 8.0 μm polyethylene terephthalate (PET) membrane (Corning) and the chambers placed in 24-well plates with media containing 10% FBS for a further 24 h. Cells were then fixed in 95% ethanol and stained using Mayer’s haematoxylin and eosin (Sigma). Images were captured using a light microscope with a 10× objective and the invading cells were counted with ImageJ software.

### Stable cell line generation

TPC1 and NIH 3T3 cell lines were transfected with VO, untagged WT PBF and C51R and R140W housed within the pcDNA3.1+ and pCI-neo vectors, respectively. After 24 h, transfected TPC1 and NIH 3T3 cells were selected with 1 mg/mL G418 (Geneticin). Single clones were isolated and expanded and the PBF expression levels of individual colonies were determined using Western blotting and TaqMan RT-PCR.

### Cell migration assays

The classical scratch wound assays were performed in triplicate in 6-well plates with NIH 3T3 cells stably expressing VO, untagged WT PBF, C51R and R140W. Wounds were created with p10 pipette tips after the cells had reached 100% confluence. Images were captured from 3 different areas within each well at 0, 4 and 6 h. The area and percentage of wound closure were analysed using ImageJ software.

### Radioiodide uptake assays

TPC1 and MCF7 cells were seeded into 24-well plates and transfected with VO, VO and NIS-MYC and NIS-MYC with untagged WT PBF, C51R or R140W. Radioiodide uptake assays were performed 48 h after transfection as described previously (Smith *et al*. 2013). Briefly, NaI (final concentration 10 µM) and 0.1 µCi ^125^I were added directly to the cell medium. After incubation at 37°C for 1 h medium was removed, and cells were washed rapidly with Hanks’ balanced salt solution. Cells were lysed in 2% sodium dodecyl sulphate, and the radioactivity of the lysate was counted for 1 min in a gamma counter. Results are given as picomoles of I^−^ per microgram of protein. To demonstrate specific uptake, the NIS inhibitor sodium perchlorate was used at 100 µM to pretreat control wells for 1 h before the addition of radioiodide.

### Colony formation assays

TPC1 cells stably expressing VO, untagged WT PBF, C51R and R140W were used in colony formation assays. The experiments were established in 6-well plates with appropriate cell density. After 14 days of incubation, colonies were stained with 0.005% crystal violet and the numbers of colonies were counted.

### Soft agar assays

NIH 3T3 stably expressing VO, untagged WT PBF, C51R and R140W were used to determine transforming ability through soft agar assays. Cells were seeded into 6-well plates, with two layers of agar; the bottom layer consisted of 0.4% sterile agar, whilst the upper layer comprised a sterile 0.35% agarose and single-cell suspension. After 14 days of incubation, colonies were stained with 0.005% crystal violet and the numbers of colonies were counted.

### Statistical analyses

Data were analysed using Student’s *t* test and Mann–Whitney *U* test for comparison between two groups of parametric and nonparametric data, respectively. Analysis of variance and Kruskal–Wallis tests were used for between-group comparisons of multiple groups of parametric and nonparametric data, respectively. Significance was taken as *P* < 0.05.

## Results

### The genomic landscape of *PBF* in cancer

**Figure 1:**
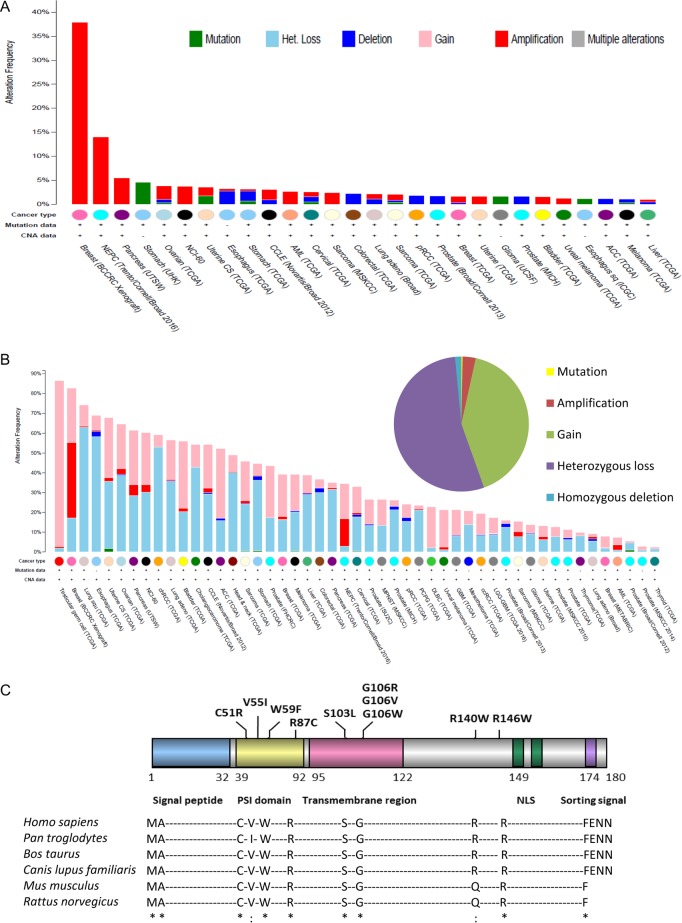
The genomic landscape of *PBF* in cancer. (A) Alteration frequency (>0%) of significant events in *PBF*, such as non-synonymous mutations, high-level amplification and homozygous deletion, in multiple human cancers in the TCGA database. (B) Alteration frequency of all events in *PBF*, including heterozygous loss and allelic gain, across all human cancer datasets with Copy Number Alteration information in the TCGA database. Inset – the distribution of all types of alteration to *PBF*. (C) Schematic diagram of PBF highlighting the first ten PBF mutations reported in the COSMIC database and their localisation within its functional domains (PSI, plexin–semaphorin–integrin; NLS, nuclear localisation signal). *Below*: amino acid conservation of the mutated residues across 6 animal species. *, identical/fully conserved amino acid; :, similar/conserved residues according to Clustal Omega analysis (http://www.ebi.ac.uk/Tools/msa/clustalo/).

Analysis of significant alterations to *PBF* within the TCGA database, such as non-synonymous mutations, high-level amplification and homozygous deletion, revealed a relatively low frequency across the multiple datasets (Fig. 1A). Amplification events were most common, particularly in breast cancer patient xenografts, neuroendocrine prostate cancers and pancreatic cancers (Fig. 1A). The inclusion of low-level copy number events such as heterozygous loss and allelic gain demonstrated a broader spectrum of alteration frequency, with 14 tumour types having more than 50% of tumours with an alteration in *PBF* copy number or mutation (Fig. 1B). Conversely, 6 tumour datasets had an alteration frequency of <10% with a further 8 datasets having no alterations at all (Fig. 1B). Of the total number of alterations observed across all datasets, only 5% were significant alterations whilst 54% were heterozygous loss and 41% were allelic gain (Fig. 1B).

The first 10 PBF mutations reported via COSMIC occurred across all functional domains of PBF, with no obvious ‘hot-spots’ other than 3 substitutions at residue G106, within the transmembrane region (Fig. 1C). Two patients shared the same G106V substitution. The 8 sites of amino acid alteration are all highly conserved across various species (Fig. 1C). Mutations were apparent primarily in colorectal cancers (*N* = 8), but also in ovary (1), lung (1) and prostate (1) tumours (Table 1).

**Table 1:** Clinical and histological data of patients with PBF mutations in various human cancers, according to the COSMIC database.

PBF mutation	Gender	Age	Tumour origin	Histology	TNM	**Vascular invasion**	Zygosity
C51R	F	66	Colon	Adenocarcinoma, poorly diff.	T4bN0M0	−	Heterozygous
V55I	M	54	Prostate	Carcinoma	Unknown	Unknown	Unknown
W59F	F	71	Lung	Adenocarcinoma, acinar type	T2aN0M0	Unknown	Unknown
R87C	F	58	Colon	Adenocarcinoma, moderately diff.	T3N0M0	−	Heterozygous
S103L	M	70	Colon	Adenocarcinoma, moderately diff.	T3N2aM0	+	Heterozygous
G106R	F	63	Ovary	Serous carcinoma	Unknown	Unknown	Heterozygous
G106V1	M	68	Colon	Adenocarcinoma, moderately diff.	TxN1bM0	−	Heterozygous
G106V2	M	45	Colon	Adenocarcinoma, moderately diff.	T3N0M0	−	Heterozygous
G106W	F	45	Colon	Adenocarcinoma, well diff.	T3N0M0	+	Heterozygous
R140W	F	58	Colon	Adenocarcinoma, moderately diff.	T3N0M0	−	Heterozygous
R146W	M	78	Colon	Adenocarcinoma, moderately to poorly diff.	T2N0M0	−	Heterozygous

F, female; M, male; M, metastasis; N, nodes; T, tumour.

### Initial biochemical characterisation of the first 10 reported mutations of PBF

**Figure 2:**
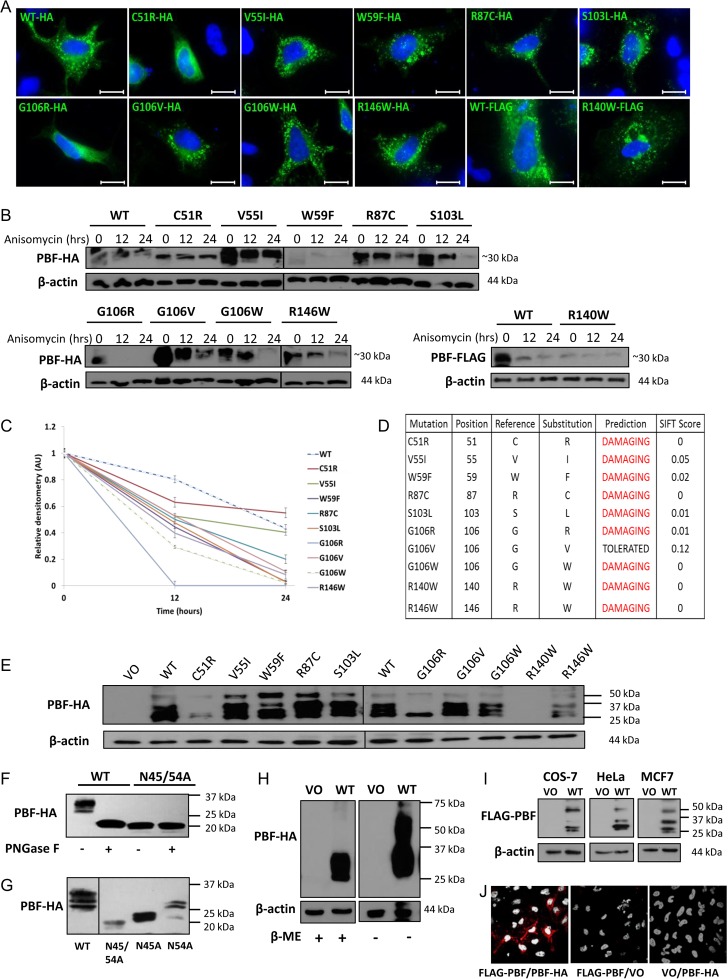
Expression, localisation and half-life of PBF mutations. (A) Subcellular localisation of each HA-tagged mutant (green) within HeLa cells using an anti-HA antibody. Blue indicates nuclear DAPI staining. As HA-tagged R140W is difficult to detect through immunofluorescent microscopy, FLAG-tagged wild-type (WT) and R140W PBF were stained using an anti-FLAG antibody. Magnification = 100×. Bars = 20 µm. (B) Anisomycin half-life assays in MCF7 cells transfected with HA-tagged WT PBF and 9 mutations and treated for 0, 12 or 24 h, with quantification of relative PBF levels shown in Western blotting (FLAG-tagged WT and R140W are shown alongside). (C) Quantification of protein stability of the ~25–37 kDa isoforms of PBF in MCF7 cells following normalisation to β-actin expression. Data presented as mean values ± s.e. (*n* = 2). (D) SIFT scores of all ten PBF mutations, where scores <0.05 are predicted to represent deleterious amino acid substitutions, which are defined as ‘damaging.’ (E) Western analysis of lysate from COS-7 cells transfected with vector only (VO), WT PBF and the 10 PBF mutants showing the expression levels and multiple forms of the HA-tagged PBF proteins. (F) PNGase F treatment of lysate from COS-7 cells transfected with WT PBF-HA results in a band at ~20 kDa and confirms N-linked glycosylation of PBF. PBF with a double substitution of N45 and N54 for alanine is also detected at ~20 kDa and cannot be modified by PNGase F confirming these are the only sites of glycosylation. (G) Western blotting of lysate from COS-7 cells transfected with HA-tagged PBF with discrete mutation of N45 and N54 suggests that both sites are glycosylated. (H) Western analysis of WT PBF-HA expressed in HeLa cells using non-reducing (minus β-mercaptoethanol (β-ME)) conditions clearly reveals the presence of a dimer ~50 kDa. (I) Western blotting demonstrating the presence of the ~50 kDa band, and therefore the ability of FLAG-PBF to form dimers, in COS-7, HeLa and MCF7 cells transfected with WT FLAG-PBF and VO control. (J) Proximity ligation assays (PLAs) demonstrating an interaction between FLAG-PBF and PBF-HA in HeLa cells (red spots indicate <30–40 nm distance between the anti-FLAG and anti-HA antibody epitopes and signify protein–protein interaction). VO co-transfections with either FLAG-PBF or PBF-HA represent negative controls.

We recapitulated all 10 mutations and examined the subcellular localisation of each mutant, HA-tagged at the C-terminus (green), within HeLa cells (Fig. 2A). HA-tagged R140W was difficult to detect through immunofluorescence (IF), so a version that was FLAG-tagged at the N-terminal end was used and compared to FLAG-tagged WT PBF. Compared with untagged PBF, PBF-HA and FLAG-PBF demonstrated no significant difference in subcellular localisation (Supplementary Fig. 1, see section on supplementary data given at the end of this article). PBF is predominantly expressed in late endosomes and at the plasma membrane (Smith *et al*. 2009, 2012, 2013). Most mutants retained a WT vesicular intracellular localisation, apart from C51R, G106R and R140W (Fig. 2A). To investigate whether these cellularly expressed PBF mutants were stable *in vitro*, we carried out protein half-life studies in MCF7 cells transfected with HA-tagged WT and mutated constructs and treated with anisomycin to inhibit new protein synthesis for 0, 12 and 24 h before harvesting. Broadly, all mutations were evident at the protein level and showed similar molecular weights to WT PBF (~30 kDa; Fig. 2B). However, although detectable through IF, W59F frequently demonstrated low expression, and R140W could not be detected via C-terminal HA tagging. Scanning densitometry to determine the stability of PBF revealed that mutations C51R and V55I, both within the PSI domain of PBF, resulted in protein stability similar to WT, with a half-life of around 22 h. FLAG-tagged R140W was also relatively stable compared to FLAG-WT PBF. In contrast, all other substitutions were markedly less stable than WT (Fig. 2C). Finally, SIFT assessment, based on sequence homology and physical properties of amino acids, demonstrated that all mutations except G106V were predicted to significantly alter protein function (Fig. 2D).

### Mutations of PBF influence post-translational processing

WT PBF-HA and the majority of the mutants were largely detected as multiple bands between 25 and 37 kDa (Fig. 2E). These bands were proposed to be differently glycosylated forms of PBF. Prediction software (http://www.cbs.dtu.dk/services/NetNGlyc/) identified two putative sites of N-linked glycosylation at residues N45 and N54. Tagging PBF either at the C-terminus with HA or near the N-terminus with FLAG was not predicted to result in different glycosylation patterns as determined via http://www.cbs.dtu.dk/services/NetNGlyc/ (data not shown), although this was not confirmed experimentally. Following treatment of lysate from COS-7 cells transfected with PBF-HA with PNGase F to remove N-linked oligosaccharides, PBF was detected at its predicted molecular weight of ~20 kDa (Fig. 2F). Double substitution of both N45 and N54 with alanine also resulted in a protein of the same size (Fig. 2F), as did treatment with the glycosylation inhibitor tunicamycin (data not shown). Mutants C51R and G106R showed a banding pattern distinctive from all others, losing all but the lowest of the glycosylated bands at ~25 kDa (Fig. 2E). Discrete mutation of N45 and N54 suggested that these lower bands relate to a form of PBF glycosylated only at N45 whilst the upper bands represent protein modified at both sites (Fig. 2G). WT PBF does not appear to be glycosylated at N54 alone (Fig. 2G). Thus, mutation of C51R and G106R appears to prevent the full processing of this glycoprotein.

Our previous unpublished studies suggest that PBF can oligomerise. Analysis of these mutants highlighted a putative dimer at ~50 kDa (Fig. 2E). Under normal reducing conditions, the dimeric form of PBF is frequently difficult to detect. However, under non-reducing conditions a PBF-HA dimer is clearly present (Fig. 2H). A 50 kDa band is also evident for the FLAG-tagged PBF protein (Fig. 2I), and PLAs clearly demonstrate an interaction between PBF-HA and FLAG-PBF (Fig. 2I) further confirming PBF dimerisation. The V55I, W59F, R87C and S103L mutations appeared to increase PBF dimerisation, which was evident even under normal reducing conditions (Fig. 2E).

### Mutations of PBF modulate cellular proliferation and subcellular localisation

**Figure 3:**
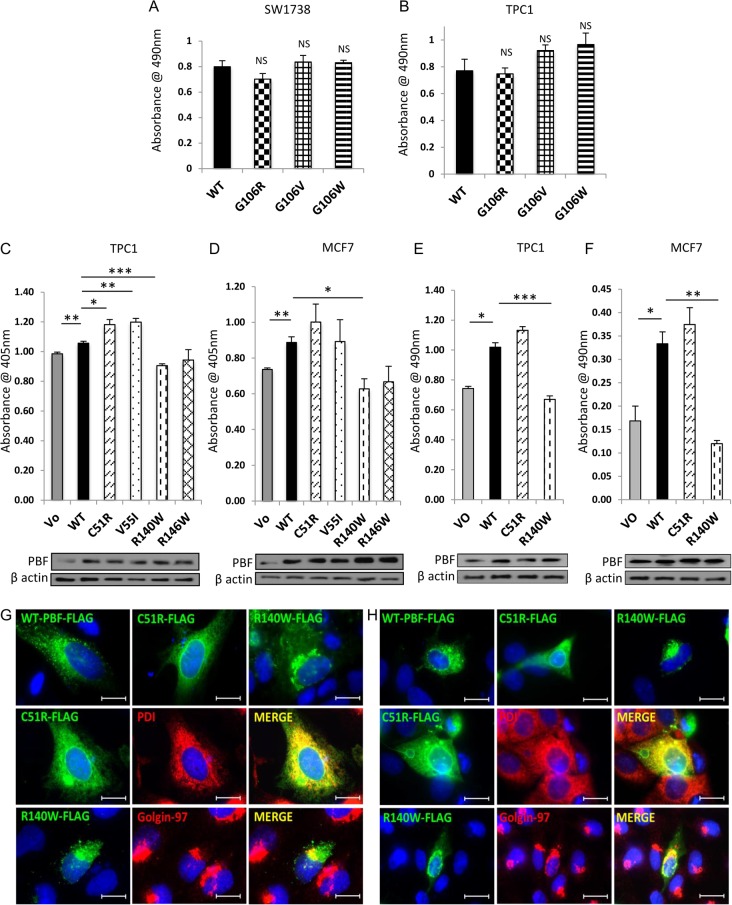
The influence of PBF mutations on thyroid and breast cell proliferation. MTS proliferation assays in SW1736 (A) and TPC1 (B) thyroid cancer cells demonstrated that 3 substitution mutations at residue G106 failed to significantly alter mitochondrial activity, compared with WT PBF, as a marker of cell number. BrdU cellular proliferation experiments in TPC1 (C) and MCF7 (D) cells transfected with VO, WT PBF, C51R, V55I, R140W and R146W mutants. MTS proliferation assays in TPC1 (E) and MCF7 (F) cells transfected with VO, WT PBF, C51R and R140W substitutions. *Beneath* – Western blots probed with anti-PBF antibody and β-actin as loading control demonstrate PBF expression to assess transfection. Data presented as mean values ± s.e. (*n* = 3). *P < 0.05, **P < 0.01, ***P < 0.001. Representative immunofluorescent microscopy of FLAG-tagged PBF and C51R and R140W mutants (green) and ER marker (anti-PDI Ab, red) or Golgi marker (anti-golgin-97 Ab, red) with subcellular co-localisation (yellow) in HeLa (G) and MCF7 (H) cells. Magnification = 100×. Bars = 20 µm.

To date, PBF function has been studied most extensively in thyroid and breast cancer cell models (Stratford *et al*. 2005, Watkins *et al*. 2010, Xiang *et al*. 2012, Read *et al*. 2014). *In vivo*, PBF can enhance thyroid cell proliferation, as evidenced by gross thyroid enlargement accompanied by a significant induction of the proliferative marker cyclin D1 in transgenic mice (Read *et al*. 2011). We therefore sought to identify whether any of the described mutations conferred a growth advantage. Given that the G106 residue carried 3 mutations (G106R, G106V and G106W), initial proliferation assays were carried out in thyroid cells transfected with WT PBF and the G106 mutants. However, all 3 mutations failed to significantly alter proliferative capacity in both SW1736 (Fig. 3A) and TPC1 cells (Fig. 3B) using MTS assays and also in BrdU incorporation experiments (data not shown). Hence residue G106 was subsequently considered not to be a hot-spot of mutational activation. Preliminary screening of all 10 mutations (data not shown) also enabled us to narrow our search to 2 mutations which increased cell turnover (C51R and V55I), and 2 mutations which repressed cell turnover (R140W and R146W) compared to WT. Thus BrdU assays, which indirectly assess DNA replication as a marker of cell division, revealed that C51R and V55I induced cell proliferation in TPC1 thyroid and MCF7 breast cells compared with WT, whilst R140W and R146W lost the pro-proliferative capacity of WT PBF (Fig. 3C and D). MTS assays, which act as a marker of cell number through indirectly measuring mitochondrial activity, revealed largely consistent findings, particularly with regard to the reduced proliferative influence of R140W (Fig. 3E and F) compared to WT PBF. For subsequent investigations we therefore confined ourselves to the 2 mutations which (i) showed the most obvious differences in cellular proliferation compared with WT PBF, (ii) were potentially damaging to protein function via SIFT, (iii) were apparent in the same tumour category (Table 1), (iv) showed altered subcellular distribution, (v) occurred at evolutionarily conserved sites, and (vi) were clearly expressed *in vitro*; C51R (N-terminal, within the PSI domain) and R140W (C-terminal, adjacent to the nuclear localisation signal). We next categorised the subcellular localisation of FLAG-tagged mutants C51R and R140W more specifically through immunofluorescent microscopy in HeLa (Fig. 3G) and MCF7 (Fig. 3H) cells. C51R was mainly localised to the endoplasmic reticulum (ER), as evidenced by its co-localisation with the ER marker PDI. R140W, by contrast, was predominantly apparent in the Golgi apparatus, where it was co-located with the Golgi marker, golgin-97 (Fig. 3G and H). Thus we selected 2 candidate PBF mutations to explore in more functional depth.

### The influence of C51R and R140W mutants on cell invasion and migration

**Figure 4:**
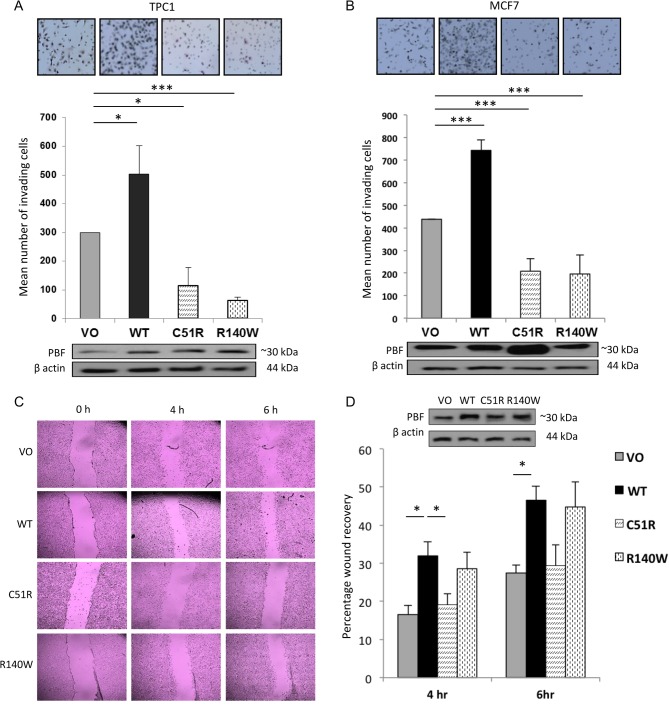
Invasive and migratory properties of C51R and R140W. 2D Boyden assay chamber cell invasion experiments in TPC1 (A) and MCF7 (B) cells transfected with VO, WT PBF, C51R and R140W mutants, with representative photomicrographs shown above. Data presented as mean values ± s.e. (*n* = 2). (C) Representative classical scratch wound assays in NIH 3T3 cells stably transfected with VO, WT PBF, C51R and R140W substitutions at 4 and 6 h, with percentages of wound recovery shown graphically in D. *Inset* – Western blotting analysis of PBF levels in the stable NIH 3T3 cells using an anti-PBF antibody. Data presented as mean values ± s.e. (*n* = 3). *P < 0.05, ***P < 0.001.

Previous studies from our group revealed that PBF significantly induces cellular invasion and migration in breast cancer (Watkins *et al*. 2010). More recent investigations have demonstrated that PBF can induce invasion and migration in multiple cancer cells including thyroid and colorectal cells (Watkins *et al*. 2016). Furthermore, these studies suggest that this occurs at least partly through interaction with cortactin (CTTN), a scaffold protein that acts predominantly at the cell periphery to promote actin polymerisation and can thus exert a potent influence upon cell movement and invasion (Watkins *et al*. 2016). Here, the impact of the PBF substitutions on cell invasion was determined through 2D Boyden chamber cell invasion experiments in TPC1 and MCF7 cells transfected with VO, WT PBF, C51R and R140W mutants. As before (Watkins *et al*. 2010), WT PBF was markedly pro-invasive compared to VO, in both TPC1 (Fig. 4A) and MCF7 (Fig. 4B) cells. However, both C51R and R140W substitutions entirely lost their invasive capacity, and in fact significantly repressed cellular invasion compared with VO control cells (Fig. 4A and B).

As cellular invasion is associated with and encompasses cell migration, we utilised classical scratch wound healing assays to measure cell migration in NIH 3T3 cells stably transfected with VO, WT PBF, C51R and R140W. WT PBF had a significant pro-migratory capacity compared to VO controls (Fig. 4C and D). In contrast, both mutants C51R and R140W lost the ability to induce wound healing compared to stable WT cells (Fig. 4C and D). Therefore, 2 mutant forms of PBF discovered in patients with cancer, one N-terminal and the other C-terminal, both lost the ability to induce cell invasion and cell migration.

### PBF mutations retain the capability to repress radioiodide uptake *in vitro*

**Figure 5:**
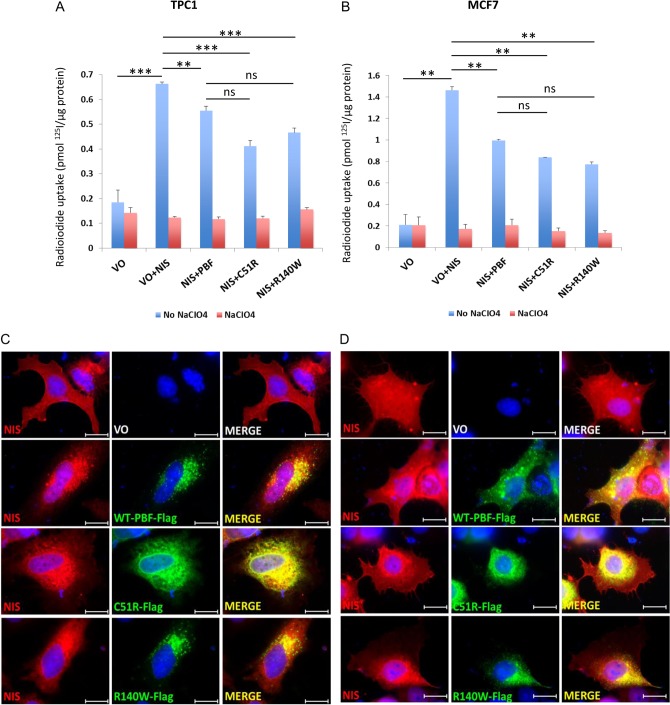
PBF mutations C51R and R140W retain the capability to repress radioiodide uptake *in vitro*. Radioiodide uptake experiments in TPC1 (A) and MCF7 (B) cells transiently transfected with VO, NIS-MYC and VO, WT PBF, C51R or R140W. Uptake was normalised to protein concentration and specific NIS-mediated uptake was demonstrated using the NIS inhibitor sodium perchlorate. Data presented as mean values ± s.e. (*n* = 3). **P < 0.01, ***P < 0.001. Immunofluorescent microscopy of NIS-MYC (red) with FLAG-tagged WT PBF, C51R or R140W (green), using the NIS and FLAG antibodies, respectively, in HeLa (C) and MCF7 (D) cells. NIS-MYC was observed predominantly at the plasma membrane with VO co-transfection and with the co-transfection of WT PBF, C51R or R140W mislocalised to intracellular vesicles, ER and Golgi, respectively, where co-localisation was observed (yellow). Magnification = 100×. Bars = 20 µm.

The best-characterised function of PBF lies in its repression of radioiodide uptake via the cellular redistribution of the sodium iodide symporter, NIS (Boelaert *et al*. 2007, Smith *et al*. 2009, 2013, Read *et al*. 2011). To further examine the impact of the PBF C51R and R140W mutations, we conducted radioiodide (^125^I) uptake assays in TPC1 and MCF7 cells co-transfected with NIS-MYC and VO, WT PBF, C51R or R140W. Transient NIS transfection induced ^125^I uptake compared to VO (Fig. 5A and B). Co-transfection with WT PBF resulted in significantly reduced uptake in both TPC1 and MCF7 cells. Radioiodide uptake was higher in MCF7 than TPC1 cells, presumably reflecting differences in transient transfection efficiencies. Interestingly, both PBF mutants retained the ability to repress radioiodide uptake in both cell lines (Fig. 5A and B). To examine this more closely, we determined the co-localisation of FLAG-tagged WT or mutated PBF with NIS-MYC through immunofluorescent microscopy in HeLa (Fig. 5C) and MCF7 cells (Fig. 5D). WT PBF co-localised with NIS predominantly in cellular vesicles, as we have reported previously (Smith *et al*. 2009, 2013). However, C51R and NIS mostly co-localised in the ER, and major subcellular co-localisation between R140W and NIS was apparent in the Golgi apparatus (Fig. 5C and D). Hence, although mutations C51R and R140W show altered subcellular localisation, they retain NIS co-localisation and preserve the ability to repress radioiodide uptake. Furthermore, these data suggest for the first time that the ability of PBF to bind NIS may not be confined to the plasma membrane and intracellular vesicles.

### C51R and R140W substitutions lose cell transforming ability compared to WT PBF

**Figure 6:**
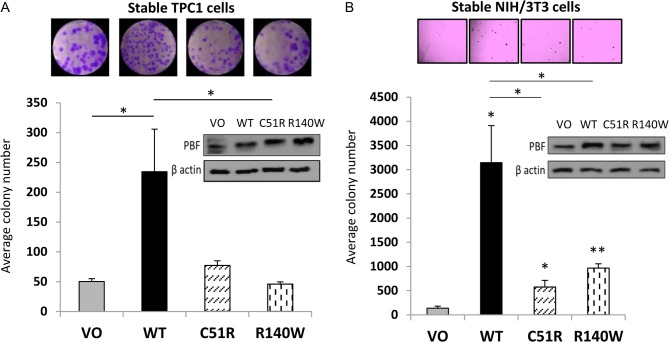
C51R and R140W substitutions lose cell transformation ability compared to WT PBF. (A) Colony formation assays in TPC1 cells stably transfected with VO, WT PBF, C51R and R140W; representative photomicrographs shown above. (B) Soft agar assays in NIH 3T3 cells stably transfected with VO, WT PBF, C51R and R140W mutants. Representative images above illustrate relative numbers of colonies. *Inset* – Western blots of PBF expression in the stable cell lines using an anti-PBF antibody. Data presented as mean values ± s.e. (*n* = 3, each with >3 replicates). *P < 0.05.

Overexpression of PBF induces cellular transformation *in vitro* (Stratford *et al*. 2005). To determine whether PBF mutants C51R and R140W retain this ability, we firstly utilised colony formation assays in TPC1 cells stably transfected with VO, WT PBF, C51R or R140W. In contrast to WT PBF, both mutants failed to induce colony formation (Fig. 6A). We further conducted soft agar experiments in NIH 3T3 cells stably transfected with VO, WT PBF, C51R or R140W mutants. WT PBF demonstrated a dramatic increase in transforming ability compared to stable VO controls (Fig. 6B), as we have reported previously (Stratford *et al*. 2005). However, mutants C51R and R140W entirely lost the ability to elicit anchorage-independent growth in soft agar assays (Fig. 6B).

Taken together, the rare PBF mutations recently reported in multiple human tumour types are unlikely to be aetiological. Although some canonical PBF functionality is retained, we propose that PBF overexpression in human tumours, rather than sequence alteration, is the more important driver of cellular transformation. Furthermore, these findings suggest that caution is required in the interpretation of whole-genome sequencing of tumours, given that even mutations in established oncogenes are frequently passenger events.

## Discussion

Recent initiatives such as COSMIC and TCGA are unearthing a myriad of mutational events in thousands of human tumours. However, only a minority of these will confer selective growth advantage and clonal expansion of the cells in which they are found. The silent majority are likely to represent the product of the unstable genetic environments which generally develop during cancer progression.

A number of statistical approaches have been utilised to help predict driver mutations and genes, based largely on protein function, mutational frequency and pathway involvement. One such statistical approach – the Driver Oncogene and Tumor Suppressor Finder – predicted that the proto-oncogene PBF/PTTG1IP may be one of 12 genes driving thyroid carcinogenesis in 326 tumour samples from TCGA (Melloni *et al*. 2014). We have previously appraised the oncogenic function of PBF in numerous studies, and we and others have reported significantly induced expression of the gene in thyroid, breast, pituitary and colorectal tumours (Boelaert *et al*. 2003, McCabe *et al*. 2003, Stratford *et al*. 2005, Watkins *et al*. 2010, Hsueh *et al*. 2013, Read *et al*. 2016). The possibility that mutations in PBF may be oncogenic led us to evaluate the functional effect of the first reported COSMIC mutations. Using PBF as a model gene to interrogate the findings of COSMIC and TCGA, we now report that even mutations in genes with known oncogenic functions do not necessarily result in growth advantage or other hallmarks of driver gene aetiology.

PBF is a widely expressed glycoprotein with a 180 amino acid sequence possessing an N-terminal signal peptide, a transmembrane domain, a bipartite nuclear localisation signal and a tyrosine-sorting signal. PBF shares no homology with any other human proteins but is well conserved among a diverse range of animal species. Having established the basic biochemistry of the first 10 mutations reported in COSMIC, we narrowed our subsequent functional studies to two potentially key substitutions: C51R and R140W. One criticism of this approach is that we may have missed genuinely oncogenic activities in the 8 other mutations which we did not pursue in depth. However, it was impractical to perform, for example, simultaneous 2D invasion assays for all 10 mutations, and initial proliferation data suggested no significant alterations in cell turnover. Our initial thought was that residue G106 might represent a hot-spot of mutational activation. However, G106V/R/W all failed to significantly enhance markers of cell turnover, and their appearance in COSMIC may therefore be coincidental. Additionally, the G106R mutation was particularly unstable, decreasing its likelihood of sustained oncogenic activity.

Currently, there are 28 reported PBF mutations in the COSMIC database, the vast majority being single amino acid substitutions. The overall incidence of PBF mutations across all cancers sequenced to date in the TCGA project is less than 0.5%. No PBF mutations have been reported in thyroid cancer; indeed the majority of mutations in this study were apparent in colorectal cancer. Additionally, there was no apparent correlation between PBF mutation status and clinical outcome in colorectal cancer. This is in contrast to previously published clinical associations in colorectal cancer, as well as in thyroid and breast neoplasia, where PBF expression significantly correlated with tumour grade and/or patient outcome (Boelaert *et al*. 2003, McCabe *et al*. 2003, Stratford *et al*. 2005, Watkins *et al*. 2010, Hsueh *et al*. 2013, Read *et al*. 2016).

One interesting facet of our *in vitro* work was that mutations abrogated certain functions of PBF (cell invasion, migration, colony formation), whilst retaining others (proliferation, radioiodide uptake repression). This sheds new light particularly into the repression of NIS function, in that C51R (predominantly restricted to the ER) and R140W (mainly in the Golgi) still co-localised with NIS and markedly inhibited radioiodide uptake. PBF binds to NIS (Smith *et al*. 2009, 2013) and, while not demonstrating an interaction between NIS and these mutants, we would now speculate that NIS:PBF interaction can occur in multiple cellular compartments, and not solely at the plasma membrane and in intracellular vesicles as previously hypothesised (Smith *et al*. 2009). In stark contrast, C51R and R140W both lost the ability to induce cell invasion, migration, colony formation and anchorage-independent growth. We suggest that these processes are all dependent upon correct subcellular localisation of PBF, particularly as the protein stability of C51R and R140W was not significantly different from WT. C51R also showed altered glycosylation. Although we frequently detect – and have now confirmed – glycosylation and dimerisation of PBF, the functional significance of these post-translational processes is not currently known. It would thus appear that glycosylation, dimerisation and subcellular localisation differentially impact on PBF’s range of *in vitro* functions, which we are currently trying to understand in more detail. One caveat here is that while we did not find that the tags used (HA, FLAG and MYC) impacted upon WT PBF processing, localisation or function, there remains the possibility that PBF mutants may be differentially ‘sensitive’ to the addition of tags. This would need to be assessed in further studies.

Importantly, in the standard biochemical assays employed here we were unable to detect any gain-of-function. Although C51R demonstrated increased cell turnover in BrdU assays, this did not translate to enhanced colony formation or anchorage-independent growth.

We therefore conclude that rare mutations in PBF are unlikely to be driver events in human cancer. Overexpression of WT PBF, which has been associated with tumour induction in xenograft models (Stratford *et al*. 2005), hyperplasia and follicular lesions in transgenic mice (Read *et al*. 2011), extramural vascular invasion in human colorectal tumours (Read *et al*. 2016), and reduced disease-specific survival in patients with thyroid tumours (Hsueh *et al*. 2013), is a more pertinent driver event. The TCGA data suggest that an increase in PBF expression could often be at least partly due to increased copy number (Fig. 1). However, the paucity of *PBF* alterations in the thyroid cancer dataset fails to explain the high levels of PBF in these tumours and suggests the presence of other, as yet unknown, mechanisms that lead to increased PBF expression. Thus, why PBF is upregulated in tumours, and exactly how this induction drives hyperplastic and neoplastic progression, remains ill defined.

## Supplementary Material

Supporting Figure 1

## Declaration of interest

The authors declare that there is no conflict of interest that could be perceived as prejudicing the impartiality of the research reported.

## Funding

This work was supported by the Faculty of Medicine Siriraj Hospital, Mahidol University, Bangkok, Thailand and the Medical Research Council (grant number RRAK16296).
