# Supplementary figures and images for: Functional consequences of the first reported mutations of the proto-oncogene PTTG1IP/PBF
Source: Endocr Relat Cancer. 2017 Jul 4;24(9):459–74. doi: 10.1530/ERC-16-0340 (PMC5551380; doi:10.1530/ERC-16-0340)

Supplementary Figure 1

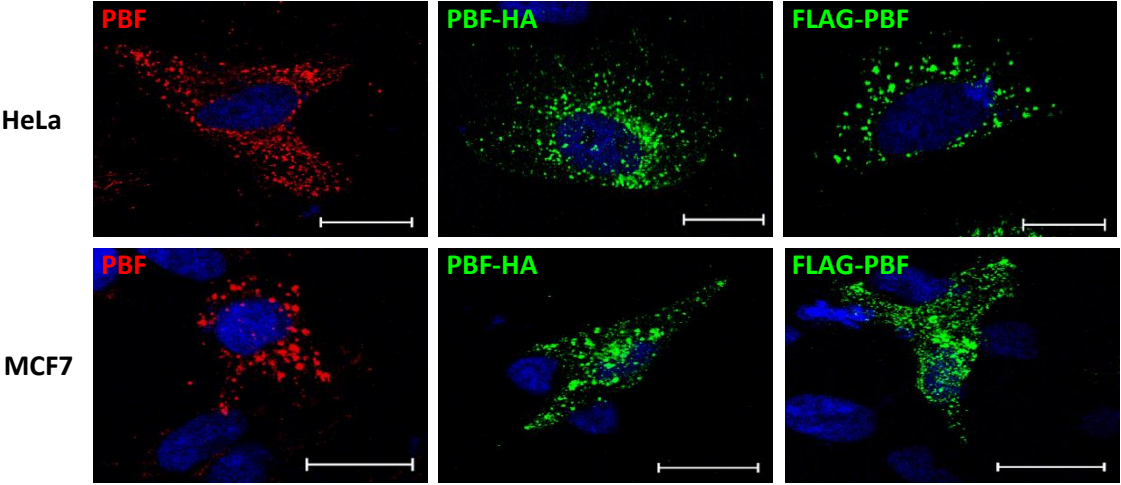

Supplement: Supporting Figure 1 [file erc-24-459-s001.pdf]
